# Ursodeoxycholic acid improves pregnancy outcome in patients with intrahepatic cholestasis during pregnancy: A protocol for systematic review and meta-analysis

**DOI:** 10.1097/MD.0000000000023627

**Published:** 2021-01-29

**Authors:** Yan Wang, Xiabiao Peng, Yongyuan Zhang, Qiuchen Yang, Yuhong Xiao, Yuezhou Chen

**Affiliations:** a Department of Gastroenterology, Boai Hospital of Zhongshan Affiliated to Southern Medical University, Zhongshan; b Reproductive and Genetic Medicine Center, The Fifth Affiliated Hospital of Sun Yat-sen University, Zhuhai, Guangdong province, China.

**Keywords:** ICP, meta-analysis, pregnancy outcome, protocol, UDCA

## Abstract

**Background:**

Intrahepatic cholestasis of pregnancy (ICP) is a common complication in the third trimester of pregnancy, which may result in premature delivery, fetal distress, stillbirth, and other adverse pregnancy outcomes. Ursodeoxycholic acid (UDCA) is a first-line treatment for ICP and has been controversial in improving adverse pregnancy outcomes. The purpose of this protocol is to systematically evaluate the effect of UDCA on pregnancy outcomes in patients with intrahepatic cholestasis during pregnancy.

**Methods:**

To search the databases PubMed, Embase, Web of Science, the Cochrane Library, CNKI, WanFang, VIP, CBMDIsc by computer, then to include randomized controlled clinical studies on UDCA for treatment of intrahepatic cholestasis during pregnancy from the establishment of the database to October 1, 2020. Two researchers independently extract and evaluate the data of the included studies, and meta-analysis is conducted on the included literatures using RevMan5.3 software.

**Results:**

This protocol evaluates the outcome of UDCA in improving ICP by incidence of postpartum hemorrhage in pregnant women preterm birth rates meconium contamination rate in amniotic fluid incidence of fetal distress scale of newborns scoring <7 in 5-min Apgar incidence of neonatal admission to neonatal intensive care unit.

**Conclusion:**

This protocol will provide an evidence-based basis for clinical use of UDCA in the treatment of intrahepatic cholestasis during pregnancy.

**Ethics and dissemination:**

Private information from individuals will not be published. This systematic review also does not involve endangering participant rights. Ethical approval was not required. The results may be published in a peer-reviewed journal or disseminated at relevant conferences.

**OSF Registration number:**

DOI 10.17605 / OSF.IO / BE67H.

## 1 Introduction

Intrahepatic cholestasis of pregnancy (ICP) is one of the complications of pregnancy middle-late, mainly for patients with skin itching and jaundice and other symptoms, and clinical inspection is usually accompanied by elevated bile acid, and liver function abnormal biochemical index.^[1,2]^ Patients with this disease have a good prognosis. In general, various clinical symptoms of ICP will subside spontaneously after delivery, but it has a serious adverse effect on the patient's fetus,^[3]^ which may cause premature delivery, amniotic fluid staining, neonatal asphyxia, sudden fetal death, intrauterine stillbirth, and other adverse pregnancy outcomes.^[4,5]^ The etiology of ICP is still unknown, and may involve genetic, environmental, hormonal, and immunological factors.^[6]^ The incidence of ICP varies greatly among different ethnic groups and regions. In Poland, the incidence is 1% to 4%, whereas in Europe, North America, and Australia, the incidence is about 1% to 2%,^[7]^ and in China, the incidence is 2.3% to 6.0%.^[8]^

Statistics have shown that although clinical treatment can improve the prognosis of ICP, there is still a high recurrence rate.^[9]^ At present, clinical treatment is mainly aimed at improving patients’ clinical symptoms and improving fetal prognosis.^[10]^ Ursodeoxycholic acid (UDCA) is a first-line drug for the treatment of ICP, characterized by safety, convenience, effectiveness, and high compliance, and widely used in clinical practice.^[11,12]^ Some studies have shown that UDCA has a certain clinical effect on improving the pregnancy outcomes of ICP patients,^[13]^ but other studies have found that UDCA cannot effectively reduce the adverse perinatal outcomes in women with ICP.^[14]^ Therefore, UDCA is controversial for improving pregnancy outcomes in patients with intrahepatic cholestasis during pregnancy. Therefore, this protocol plans to include a randomized controlled trial (RCT) of UDCA for ICP published before October 1, 2020. Meta-analysis will be conducted with RevMan5.3 software to provide an evidence-based basis for clinical use of UDCA for ICP.

## 2 Methods

### 2.1 Protocol register

This protocol of systematic review and meta-analysis has been drafted under the guidance of the preferred reporting items for systematic reviews and meta-analysis protocols (PRISMA-P). In addition, it has been registered on the open science framework (OSF) on November 5, 2020 (registration number: DOI 10.17605 / OSF.IO / BE67H).

### 2.2 Ethical approval

For patients are not recruited for this study, approval from an Ethics Committee is not required.

### 2.3 Eligibility criteria

#### 2.3.1 Research type

We will collect all UDCA RCTs for the treatment of ICP, which are unlimited in magazine, publication year, the region, and blind method, but only in Chinese and English.

#### 2.3.2 Research subjects

For patients with definite diagnosis of ICP, the diagnostic criteria are referred to the Guidelines for diagnosis and Treatment of Intrahepatic cholestasis of Pregnancy (2015),^[15]^ and there are no restrictions on nationality, race, age, onset time, and other conditions.

#### 2.3.3 Intervention measures

The intervention measures for the treatment group are UDCA alone or the control group with UDCA. The control group is treated with conventional western medicines such as placebo, low-molecular-weight heparin (LMWH), dexamethasone, S-adenosine methionine, adenosine succinate, polyene phosphatidylcholine, and so on. Dosage and course of treatment in both groups are not limited.

#### 2.3.4 Outcome Indicators

Outcome indicators are : incidence of postpartum hemorrhage in pregnant women; preterm birth rate; meconium contamination rate in amniotic fluid; incidence of fetal distress; newborns with 5-minute Apgar score <7 ratio^[16]^; in newborn intensive care unit rates.

### 2.4 Exclusion Criteria

1. Repeatedly published papers, the one with the most complete data;
2. Articles published in abstracts or without full-text articles, and the data cannot be obtained after contacting the author;
3. Studies with obvious data errors;
4. The treatment group adopted traditional Chinese medicine therapy, such as acupuncture and moxibustion, Chinese herbal compound, among others.

### 2.5 Retrieval Strategy

*UDCA* and *ICP* are used as Chinese search terms and retrieved in Chinese databases, including CNKI, WanFang, VIP, and China Biomedical Database. Taking *UDCA*, *ICP*, *pregnancy cholestasis*, and *cholestasis in pregnancy* as search terms, the search is conducted in the English database, including PubMed, EMBASE, Web of Science, the Cochrane Library, and so on. All the domestic and foreign RCTs of UDCA in the treatment of ICP are collected from the time of database establishment to October 1, 2020. Take PubMed as an example, and the retrieval strategy is shown in Table 1.

**Table 1:** Search strategy in PubMed database.

Number	Search terms
#1	Intrahepatic cholestasis of pregnancy [MeSH]
#2	Intrahepatic cholestasis of pregnancy [Title/Abstract]
#3	pregnancy cholestasis [Title/Abstract]
#4	cholestasis in pregnancy [Title/Abstract]
#5	Recurrent intrahepatic cholestasis of pregnancy [Title/Abstract]
#6	Pregnancy related cholestasis [Title/Abstract]
#7	Pregnancy-Related Cholestasis [Title/Abstract]
#8	Obstetric Cholestasis [Title/Abstract]
#9	Familial intrahepatic cholestasis of pregnancy [Title/Abstract]
#10	#1 OR #2 OR #3 OR #4 OR #5 OR#6 OR #7 OR #8 OR #9
#11	ursodesoxycholic acid [MeSH]
#12	ursodesoxycholic acid [Title/Abstract]
#13	Ursacholic Acid[Title/Abstract]
#14	Ursodiol[Title/Abstract]
#15	UDCA [Title/Abstract]
#16	#11 OR #12 OR #13 OR #14 OR #15
#17	pregnancy outcome [MeSH]
#18	pregnancy outcome [Title/Abstract]
#19	obstetric outcome [Title/Abstract]
#20	maternal outcome [Title/Abstract]
#21	fetal outcome [Title/Abstract]
#22	perinatal outcome [Title/Abstract]
#23	#17 OR #18 OR #19 OR #20 OR #21 OR #22
#24	#10 AND #16 AND #23

### 2.6 Data filtering and extraction

According to the inclusion and exclusion criteria, 2 researchers use the EndNoteX7 literature management software to check and discard. By reading the titles and abstracts of all the literatures, the full text of the literatures that basically meet the inclusion criteria are closely read, and then according to the exclusion criteria, the literatures that do not meet the inclusion criteria are further excluded, and cross-checking is carried out. If there are studies that are difficult to determine whether to be included or not, they are evaluated by a third-party researcher. Excel2013 software is also used to extract the following data from studies that met the inclusion and exclusion criteria: the first author of the literature and the year of publication; basic information of the subjects: age, sex, course of disease, and so on; intervention methods of treatment group and control group: such as the way of administration, dose; relevant outcome indicators; evaluation factors of literature bias risk.

### 2.7 Literature quality evaluation

**Figure 1:**
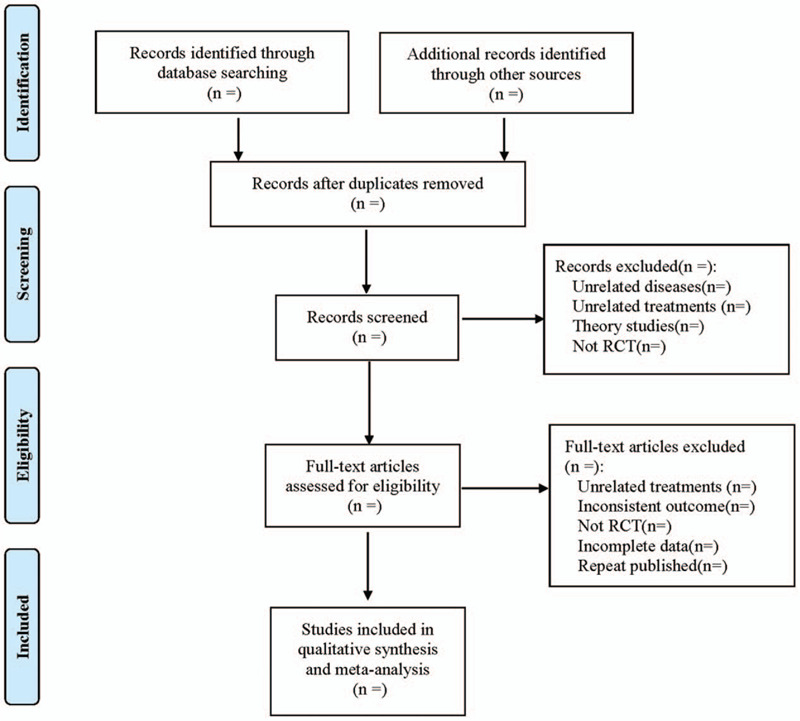
Flow diagram.

Using RevMan5.3 software built-in bias risk assessment tool (the Cochrane collaboration 's tool for assessing risk of bias) risk of bias assessment included in the study. Seven aspects of Random Sequence Generation, Allocation Concealment, Blinding of participants and Personnel, Blinding of outcome Assessment, Incomplete outcome Data, Selective Reporting and Other bias are given by the 2 researchers according to the included literatures cross-check the judgment of low risk, accountability risk, and high risk, respectively. Differences need to be discussed, and if consensus cannot be reached, it will be left to third-party researchers to discuss and resolve. The literature selection process is shown in Figure 1.

### 2.8 Statistical analysis

RevMan5.3 software is used to analyze the data of all literatures. Dichotomous variables are expressed by relative risk (RR). If the instrument and unit of measurement are identical, the weighted average (WMD) is used; if the instrument or unit of measurement is inconsistent, the standardized mean difference (SMD) is used as the effect. Heterogeneity is tested by *χ*^2^ and *I*^*2*^ values. If *P* ≥ 0.1 and *I*^*2*^ ≤ 50%, it means that there is no obvious heterogeneity in the included studies, and a fixed-effect model is used for meta-analysis. If *P* < 0.1 and *I*^*2*^ > 50%, it means that there is significant heterogeneity in the study. The sources of heterogeneity will be identified by subgroup analysis and sensitivity analysis. In the absence of significant clinical and methodological heterogeneity, random-effects models are used for analysis. If clinical heterogeneity is too obvious and subgroup analysis is not possible, only descriptive analysis will be performed. *P* < 0.05 is considered statistically significant.

#### 2.8.1 Missing data processing

If there are missing data in the article, contact the author via email for additional information. If the author cannot be contacted or the author has lost relevant data, descriptive analysis is performed or the study is excluded.

#### 2.8.2 Subgroup analysis

According to the occurrence time of ICP, subjects are divided into early ICP subgroup and late ICP subgroup. And according to the number of pregnancies, subjects are divided into the first pregnancy and the second pregnancy for subgroup analysis. Subgroup analysis is carried out according to the different western medicine used in the control group, such as UDCA versus S-adenosine (SAMe) group; UDCA versus placebo group; UDCA versus dexamethasone group; UDCA versus LMWH; UDCA versus adenosine succinate; UDCA versus polyene phosphatidylcholine, and so on.

#### 2.8.3 Sensitivity analysis

To judge the stability of outcome index, the sensitivity analysis of each outcome index was carried out by one-by-one elimination method.

#### 2.8.4 Evaluation of publication bias

Funnel plots were to be used to assess publication bias if >10 studies were included in an outcome measure. Moreover, Egger and Begg test were used for the evaluation of potential publication bias.

#### 2.8.5 Evidence quality evaluation

The Grading of Recommendations Assessment, Development, and Evaluation (GRADE) will be used to assess the quality of evidence. It contains 5 domains (bias risk, consistency, directness, precision, and publication bias). And the quality of evidence will be rated as high, moderate, low, and very low.

## 3 Discussion

ICP is a common pregnancy-specific liver disease,^[17]^ and maternal serum total bile acid level is the most important serological indicator for the diagnosis and monitoring of ICP.^[18,19]^ At present the specific pathogenesis of ICP is unclear; however, it has been shown to be associated with significantly elevated estrogen levels in pregnant women during pregnancy. Estrogen levels can lead to Na + -k + ATPase activity decline, and thus affects the energy supply, leading to bile acid metabolism disorder. Estrogen can also be through combined with estrogen receptors on the surface of the liver cells to affect liver cell protein synthesis, causing bile reflux, leading to hepatic lobule cholestasis within.^[20–22]^ Bile acid is released into the blood, the blood cholic acid concentration increased, deposited in the placental villi gap, thereby destroying the placental blood perfusion, resulting in the lack of fetal oxygen supply. In severe cases, the fetus may die of hypoxia.^[23,24]^ At the same time, the high concentration of bile acid in the blood of ICP patients will lead to the obstruction of bile acid excretion in the fetus, causing hypercholic acidemia, vasospasm, decreased blood flow, less oxygen exchange, fetal distress, and damage of fetal viscera function.^[25,26]^ High bile acid levels can also lead to a disturbance in fetal steroid metabolism, resulting in high dehydroepiandrosterone expression and the formation of large amounts of estradiol through the placenta, leading to preterm labor.^[27,28]^ In addition, too much bile acid in the fetus can stimulate intestinal smooth muscle contraction, increase intestinal peristalsis, and defecate frequently into the amniotic fluid, eventually resulting in amniotic fluid pollution.^[29–31]^ ICP is closely related to maternal fetal immune dysfunction and immune response imbalance.^[32,33]^ At present, the main clinical principle for the treatment of ICP is to protect the liver and gallbladder, improve the signs and living indicators of pregnant women, and thus improve the pregnancy outcome.^[34,35]^ Therefore, it is very important to optimize liver function and improve maternal and infant outcomes.

UDCA is a commonly used drug for the clinical treatment of ICP.^[36]^ ICP is treated through the following mechanisms: replace the endogenous bile acid on the liver membrane of patients, effectively increase the transfer of bile acid in liver cells, reduce the deposition of bile acid, and effectively reduce the content of bile acid in blood^[37]^; Reduce the gastrointestinal reabsorption of hydrophobic cholic acid, reduce the content of hydrophobic cholic acid in bile duct, and reduce the cytotoxic effect of hydrophobic cholic acid^[38]^; it has an antagonistic effect on bile acid-induced cell apoptosis, reduces the transport permeability of mitochondrial membrane, promotes its active transport function, enhances the secretion of hepatobiliary duct, and improves the transport capacity of transporters on liver cell membrane^[39]^; inhibit progesterone vulcanization metabolism synthesis, prevent liver from stress injury and apoptosis, remove bile salts formed by bile acid deposition, and induce fetal hepatobiliary system maturity^[40]^; inhibit the activity of immune factors such as interleukin-2 and INF-, reduce the inflammatory necrosis and apoptosis of hepatocytes induced by toxic T cells, and reduce the injury of hepatocytes.^[41]^ Although UDCA has not been officially endorsed or recommended, its teratogenic effects have never been reported, and a recent study concluded that its use is safe and effective.^[42]^

Therefore, an in-depth study of UDCA is needed to provide evidence-based evidence for clinicians using UDCA to treat ICP. However, this study also has some limitations. Since the included studies are limited to Chinese and English, reports or studies in other languages may be taken into account. It is expected that further studies with large samples and high-quality randomized controlled trials will be conducted to provide more evidence support for the clinical application of UDCA.

## Author contributions

**Data curation:** Yan Wang, Xiabiao Peng.

**Funding acquisition:** Yuezhou Chen.

**Resources:** Yongyuan Zhang, Qiuchen Yang.

**Software:** Yuhong Xiao.

**Supervision:** Yuezhou Chen.

**Writing – original draft:** Yan Wang, Xiabiao Peng.

**Writing – review & editing:** Yan Wang, Xiabiao Peng, Yuezhou Chen.

## Abbreviations

Abbreviations: CBMDIsc = CD ROM database of Chinese Biomedical Literature, CNKI = China National Knowledge Infrastructure, EMBASE = Excerpta Medica dataBASE, ICP = intrahepatic cholestasis of pregnancy, LMWH = low-molecular-weight heparin, PRISMA-P = Meta-analysis Protocols, RCT = randomized controlled trial, RR = relative risk, SAMe = S-adenosine, SMD = standardized mean difference, UDCA = ursodeoxycholic acid, VIP = China Science and Technology Journal Database, WMD = weighted average.
